# Supplementary material for: An extended motif in the SARS-CoV-2 spike modulates binding and release of host coatomer in retrograde trafficking
Source: Commun Biol. 2022 Feb 8;5:115. doi: 10.1038/s42003-022-03063-y (PMC8825798; doi:10.1038/s42003-022-03063-y)
Supplement: Supplementary file 1 — Supplementary Information (new) [file 42003_2022_3063_MOESM1_ESM.pdf]

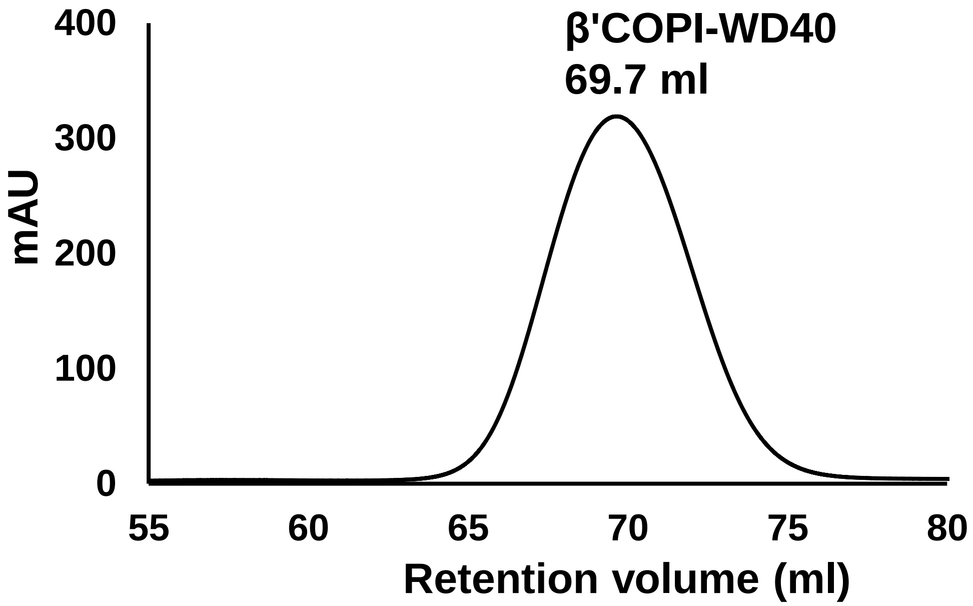

Supplementary Figure 1: Purification of β’-COPI-WD40 domain. SEC analysis of β’COPI-WD40 protein showing a single monodisperse peak. A HiLoad Superdex 75 16/600 chromatography column was used for this analysis.

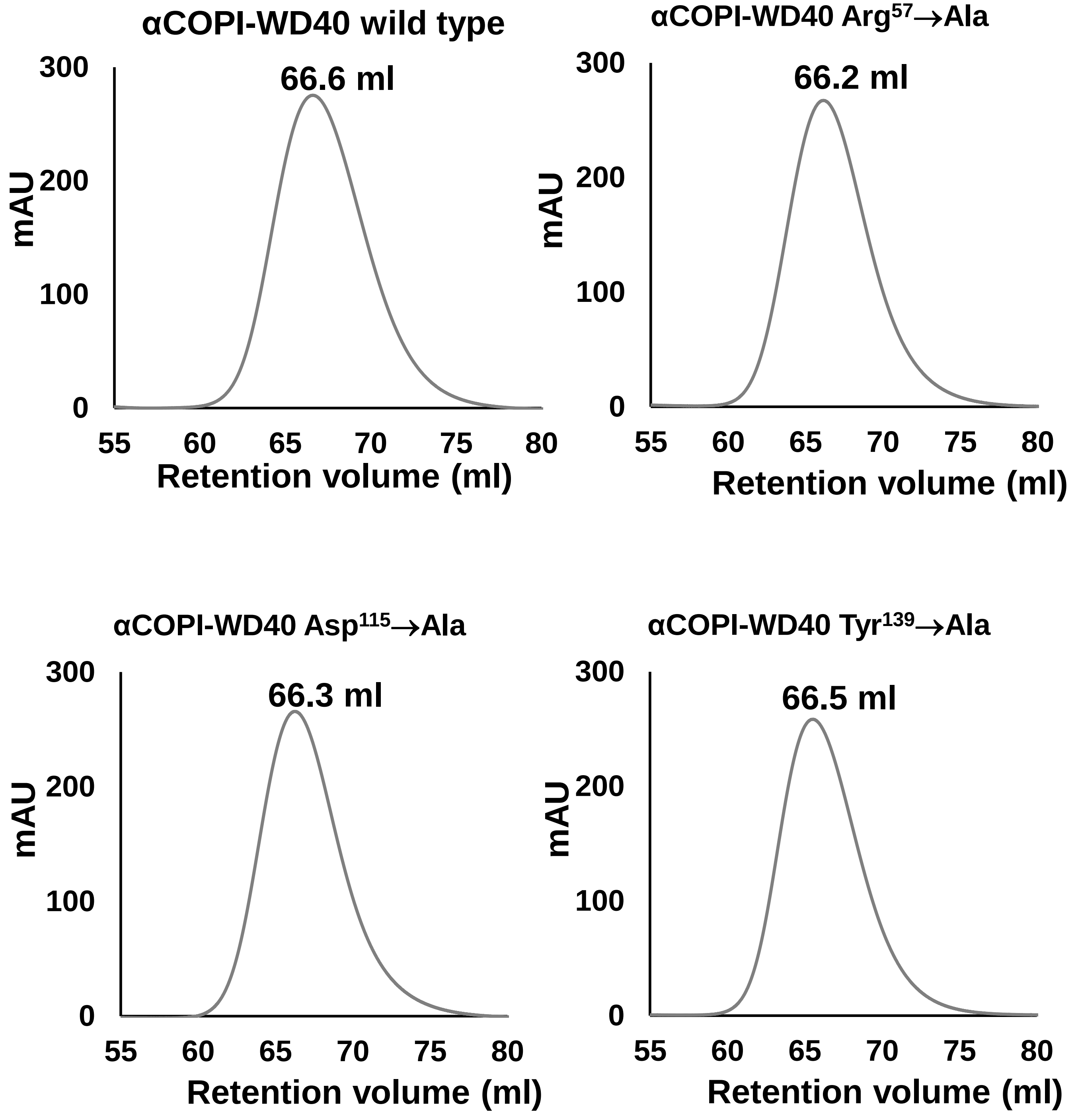

Supplementary Figure 2: Preparative SEC analysis of αCOPI-WD40 domain and mutants Arg[57]→Ala, Asp[115]→Ala, and Tyr[139] →Ala mutants. A HiLoad Superdex 75 16/600 chromatography column was used for this analysis.

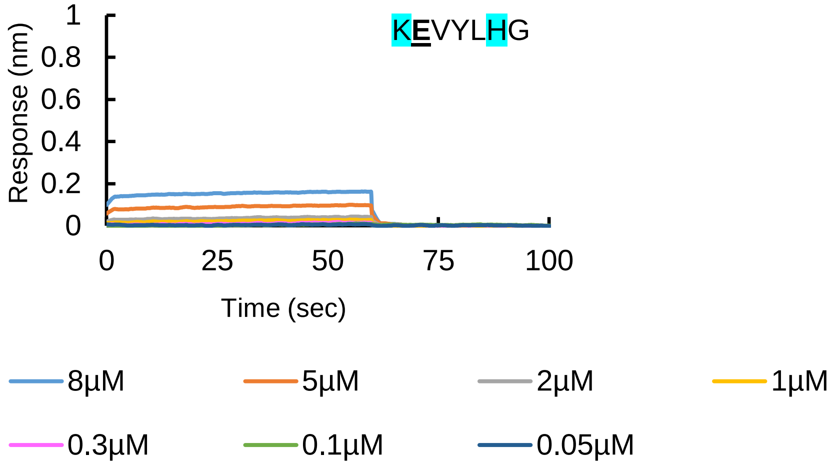

Supplementary Figure 3: BLI analysis of a spike hepta-peptide with a scrambled sequence containing the Thr[1273]→Glu mutation. This analysis shows weak binding of αCOPI-WD40 domain to this peptide. The color code corresponding to the αCOPI-WD40 concentrations is given at the bottom of the figure.

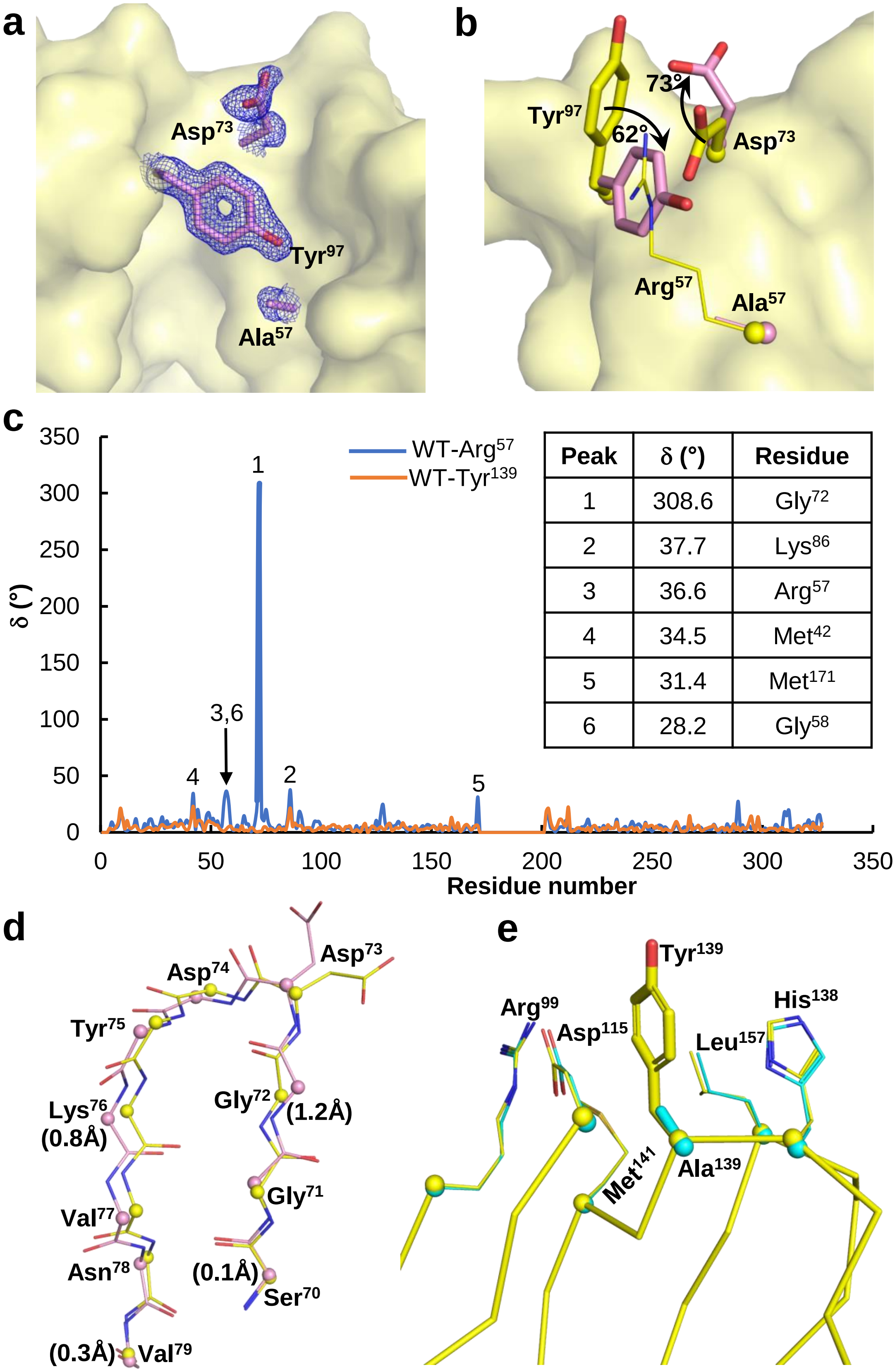

Peak	δ (°)	Residue
1	308.6	Gly72
2	37.7	Lys86
3	36.6	Arg57
4	34.5	Met42
5	31.4	Met171
6	28.2	Gly58

Supplementary Figure 4: Crystallographic analysis αCOPI-WD40 Arg^{57}→Ala and Tyr^{139}→Ala mutants. (a) Electron density around the side chains of residues Ala^{57}, Asp^{73}, and Tyr^{97} (blue mesh, 2Fo-Fc map contoured at 1.0 σ) in the structure of the Arg^{57}→Ala mutant. For simplicity, other residues are shown as a yellow surface. **(b)** Conformational changes in αCOPI-WD40 caused by Arg^{57}→Ala mutation. The mutant and wild type αCOPI-WD40 structures are shown in pink and yellow as the primary colors, respectively. The Arg^{57}→Ala mutation generates a cavity in αCOPI-WD40. The nearby Tyr^{97} residue side chain rotates into this cavity. This is accompanied by the outward rotation of the Asp^{73} side chain, which is bonded to Arg^{57} in the wild type structure. **(c)** An analysis of differences in

Supplementary Figure 4 (contd): main chain conformation between the wild type and mutant αCOPI-WD40 crystal structures. The difference in Ramachandran angles was calculated for each residue, ($\delta=\sqrt{((\psi_{WT}-\psi_m)^2+(\phi_{WT}-\phi_m)^2)}$), where ($\psi_{WT}$, ϕ_{WT}) and (ψ_m, ϕ_m) are Ramachandran angles for wild type and each mutant crystal structure. This analysis shows larger conformational changes in the $Arg^{57}\rightarrow Ala$ mutant (blue) than in $Tyr^{139}\rightarrow Ala$ mutant (orange). The top six peaks are highlighted. Peak 1 corresponds to a main chain rearrangement coincident with an outward movement of Asp^{73} as shown in panel **(d)**. Cα atoms are shown as spheres in panel **(d)**. Upto 1.2Å and 0.8Å shifts in the Cα atoms are observed for Gly^{72} and Lys^{76} respectively, between the wild type and mutant structures. The intervening residues demonstrate substantial conformational rearrangement of the main chain. **(e)** In contrast, the αCOPI-WD40 $Tyr^{139}\rightarrow Ala$ mutant structure (primary color cyan) shows limited changes from the wild type structure (primary color yellow).

Supplementary Table 1: Crystallographic data and refinement statistics

**Structure**	**αCOPI-WD40 WT**	**αCOPI-WD40 Arg^{57}→Ala**	**αCOPI-WD40 Tyr^{139}→Ala**
PDB ID	7S22	7S16	7S23
X-ray Source	23-ID-D	AMX 17-ID-1	AMX 17-ID-1
Wavelength (Å)	1.03	0.92	0.92
Temperature (K)	100	100	100
Space group	$P\ 1\ 2_1\ 1$	$P\ 1\ 2_1\ 1$	$P1\ 2_1\ 1$
Unit cell (Å, º)	a= 36.99, b=171.82, c=71.42; α=γ=90, β=99.57	a=35.87, b=56.71, c=70.55; α=γ=90, β=99.37	a=37.31, b=171.55, c=71.28; α=γ=90, β=99.71
Resolution (Å)	44.4-1.8 (1.78-1.75)	69.6-1.2 (1.26– 1.24)	171.6 -1.5 (1.52-1.49)
$^{a}R_{merge}$ (%)	9.2 (54.7)	5.4 (57.9)	8.6(71.9)
$\langle I/\sigma(I) \rangle$	8.3 (2.2)	11.5 (1.6)	5.0 (1.1)
CC1/2 (%)	98.1 (65.3)	99.8 (66.3)	99.6 (67.0)
No. of reflections	196291 (10446)	319698 (6270)	502939 (25265)
No. of unique reflections	85435 (4461)	74665(2292)	140251 (6840)
Completeness (%)	97.0 (95.4)	94.2 (58.9)	97.9 (96.8)
Redundancy	2.3 (2.3)	4.3 (2.7)	3.6 (3.7)
**Refinement Statistics**			
Resolution (Å)	44.48-1.75 (1.79-1.75)	43.97-1.24 (1.26-1.24)	65.02-1.49 (1.51-1.49)
No. of reflections (F>0) used in refinement	81123 (2645)	74567 (1653)	140153 (4335)
$^{b}R_{work}$ (%)	17.3	13.5	14.0
$^{c}R_{free}$ (%)	21.4	15.9	18.4
RMS bond length (Å)	0.007	0.005	0.005
RMS bond angle (°)	0.938	0.910	0.696

Overall B value ($Å^2$)	21.1	21.8	21.7
**Ramachandran Plot Statistics[d]**			
Residues	924	321	923
Favored (%)	96.1	95.6	95.8
Allowed (%)	3.8	4.4	4.2
Disallowed (%)	0.1	0.0	0.0

[a]R_{merge} = [∑h∑i|Ih – Ihi|/∑h∑iIhi] where Ih is the mean of Ihi observations of reflection h. Numbers in parenthesis represent highest resolution shell. [b]R_{work} and [c]R_{free} = ∑||Fobs| - |Fcalc|| / ∑|Fobs| x 100 for 95% of recorded data (R_{work}) or 5% data (R_{free}). [d]From MolProbity(Williams et al., 2018).

Supplementary Table 2: C-terminal sequence of predicted human membrane proteins with K-x-H-x-x or K-x-K-x-x motif

UNIPROT ID	Protein Name	C-terminal Sequence
Q9NVV5	Androgen-induced gene 1 protein	KPKLE
Q9BVK2	Probable dolichyl pyrophosphate Glc1Man9GlcNAc2 alpha-1,3-glucosyltransferase	KTKKQ
Q9NW15	Anoctamin-10	KEKAT
Q9P241	Phospholipid-transporting ATPase VD	KGKES
O15342	V-type proton ATPase subunit e 1	KYHWP
Q6UW56	All-trans retinoic acid-induced differentiation factor	KAKTS
Q5SY80	Cation channel sperm-associated protein subunit epsilon	KRKKN
Q96A33	PAT complex subunit CCDC47	KVKAM
P27544	Ceramide synthase 1	KDKRF
Q99675	Cell growth regulator with RING finger domain protein 1	KPKTL
Q9NZ45	CDGSH iron-sulfur domain-containing protein 1	KKKET
Q8N5K1	CDGSH iron-sulfur domain-containing protein 2	KKKEV
Q9ULY5	C-type lectin domain family 4 member E	KGKSL
Q6UXB4	C-type lectin domain family 4 member G	KRHNC
P39656	Dolichyl-diphosphooligosaccharide--protein glycosyltransferase 48 kDa subunit	KEKSD
Q6IAN0	Dehydrogenase/reductase SDR family member 7B	KSKNS
Q8NFT8	Delta and Notch-like epidermal growth factor-related receptor	KTKDL
Q15125	3-beta-hydroxysteroid-Delta(8),Delta(7)-isomerase	KSKKN
Q9BW60	Elongation of very long chain fatty acids protein 1	KVKAN
Q9HB03	Elongation of very long chain fatty acids protein 3	KTKSQ
Q9GZR5	Elongation of very long chain fatty acids protein 4	KAKGD
A1L3X0	Elongation of very long chain fatty acids protein 7	KNKDN
Q96A26	Protein FAM162A	KAKTE
Q96ND0	Protein FAM210A	KKKVE
Q9NYL4	Peptidyl-prolyl cis-trans isomerase FKBP11	KSKKK
Q96MZ0	Ganglioside-induced differentiation-associated protein 1-like 1	KKKYI
Q9P035	Very-long-chain (3R)-3-hydroxyacyl-CoA dehydratase 3	KKKIH
Q5VWC8	Very-long-chain (3R)-3-hydroxyacyl-CoA dehydratase 4	KKKKM
Q9HCP6	Protein-cysteine N-palmitoyltransferase HHAT-like protein	KEKPE
Q53GQ0	Very-long-chain 3-oxoacyl-CoA reductase	KTKKN
P37059	17-beta-hydroxysteroid dehydrogenase type 2	KKKAT
P14060	3 beta-hydroxysteroid dehydrogenase/Delta 5-->4-isomerase type 1	KSKTQ
P26439	3 beta-hydroxysteroid dehydrogenase/Delta 5-->4-isomerase type 2	KSKTQ
O15503	Insulin-induced gene 1 protein	KPHSD
Q9Y5U4	Insulin-induced gene 2 protein	KSHQE
Q96N16	Janus kinase and microtubule-interacting protein 1	KLKFM
Q86W47	Calcium-activated potassium channel subunit beta-4	KRKFS
Q643R3	Lysophospholipid acyltransferase LPCAT4	KQKGD
Q6ZNC8	Lysophospholipid acyltransferase 1	KRKTD
Q96T53	Ghrelin O-acyltransferase	KHKCN
Q8NBP5	Major facilitator superfamily domain-containing protein 9	KLKSE
Q53F39	Metallophosphoesterase 1	KRKTR
P39210	Protein Mpv17	KAHRL
Q969V3	Nicalin	KAKTQ
Q9Y266	Nuclear migration protein nudC	KAKFN
P47890	Olfactory receptor 1G1	KIHSP
Q96R27	Olfactory receptor 2M4	KRKLI

Q8NGI8	Olfactory receptor 5AN1	KRKCC
A6NM76	Olfactory receptor 6C76	KKKKH
Q8NGZ6	Olfactory receptor 6F1	KWKGK
Q9HC56	Protocadherin-9	KEHQL
O75192	Peroxisomal membrane protein 11A	KLKTR
Q96FM1	Post-GPI attachment to proteins factor 3	KFKLD
O95427	GPI ethanolamine phosphate transferase 1	KSHFM
O60486	Plexin-C1	KCKWM
Q969W9	Protein TMEPAI	KGHPL
Q16799	Reticulon-1	KRHAE
O75298	Reticulon-2	KAKAE
O95197	Reticulon-3	KKKAE
Q9NQC3	Reticulon-4	KRKAE
Q9NTJ5	Phosphatidylinositol-3-phosphatase SAC1	KEKID
Q9UI33	Sodium channel protein type 11 subunit alpha	KVHCD
P35498	Sodium channel protein type 1 subunit alpha	KAKGK
Q96BI1	Solute carrier family 22 member 18	KDKVR
P78381	UDP-galactose translocator	KVKGS
Q9NXE4	Sphingomyelin phosphodiesterase 4	KLHQP
P43308	Translocon-associated protein subunit beta	KTKKN
Q9P246	Stromal interaction molecule 2	KKKSK
O15260	Surfeit locus protein 4	KKKEW
O15533	Tapasin	KKKAE
P57738	T-cell leukemia translocation-altered gene protein	KTHRE
Q6UX40	Transmembrane protein 107	KKKPF
A0PK00	Transmembrane protein 120B	KTKQP
Q9H6L2	Transmembrane protein 231	KEHLS
Q53FP2	Novel acetylcholine receptor chaperone	KVKVS
Q5BJD5	Transmembrane protein 41B	KQKFE
Q5BJF2	Sigma intracellular receptor 2	KRKKK
Q15629	Translocating chain-associated membrane protein 1	KEKSS
Q8N609	Translocating chain-associated membrane protein 1-like 1	KEKSS
Q15035	Translocating chain-associated membrane protein 2	KLKSP
O60858	E3 ubiquitin-protein ligase TRIM13	KYKLL
Q9HCX4	Short transient receptor potential channel 7	KGKDI
Q9GZZ9	Ubiquitin-like modifier-activating enzyme 5	KMKNM
P22309	UDP-glucuronosyltransferase 1A1	KSKTH
Q9HAW8	UDP-glucuronosyltransferase 1A10	KSKTH
P35503	UDP-glucuronosyltransferase 1A3	KSKTH
P22310	UDP-glucuronosyltransferase 1A4	KSKTH
P35504	UDP-glucuronosyltransferase 1A5	KSKTH
P19224	UDP-glucuronosyltransferase 1-6	KSKTH
Q9HAW7	UDP-glucuronosyltransferase 1A7	KSKTH
Q9HAW9	UDP-glucuronosyltransferase 1A8	KSKTH
O60656	UDP-glucuronosyltransferase 1A9	KSKTH
P36537	UDP-glucuronosyltransferase 2B10	KGKRD
O75310	UDP-glucuronosyltransferase 2B11	KGKRD
P54855	UDP-glucuronosyltransferase 2B15	KKKRD
O75795	UDP-glucuronosyltransferase 2B17	KKKRD
Q9BY64	UDP-glucuronosyltransferase 2B28	KGKRD
P06133	UDP-glucuronosyltransferase 2B4	KGKRD
P16662	UDP-glucuronosyltransferase 2B7	KGKND
Q6NUS8	UDP-glucuronosyltransferase 3A1	KVKKT
Q3SY77	UDP-glucuronosyltransferase 3A2	KVKET
Q9BQB6	Vitamin K epoxide reductase complex subunit 1	KAKRH

Q8NB15	Zinc finger protein 511	KTKQC
P58397	A disintegrin and metalloproteinase with thrombospondin motifs 12	KSKEL
P20851	C4b-binding protein beta chain	KAKLL
P78556	C-C motif chemokine 20	KVKNM
Q8WUJ3	Cell migration-inducing and hyaluronan-binding protein	KKKKL
O43927	C-X-C motif chemokine 13	KRKIP
Q30KQ7	Beta-defensin 113	KLHQK
P0DP74	Beta-defensin 130A	KGKSP
P0DP73	Beta-defensin 130B	KGKSP
Q13609	Deoxyribonuclease gamma	KSKRS
P00740	Coagulation factor IX	KTKLT
P11150	Hepatic triacylglycerol lipase	KRKIR
Q8N1E2	Lysozyme g-like protein 1	KRHGF
O95631	Netrin-1	KCKKA
Q9UKZ9	Procollagen C-endopeptidase enhancer 2	KNKQC
P01270	Parathyroid hormone	KAKSQ
P07225	Vitamin K-dependent protein S	KTKNS

Supplementary Table 3: Frequency of residues in K-x-H(K)-x-x

	Residue position in dibasic motif K-x-H(K)-x-x				
	Position 1	Position 2	Position 3	Position 4	Position 5
Amino Acid					
Ala	0	9	0	8	1
Arg	0	11	0	9	3
Asn	0	2	0	5	7
Asp	0	2	0	3	13
Cys	0	2	0	3	3
Glu	0	7	0	5	11
Gln	0	2	0	6	5
Gly	0	9	0	5	0
His	0	1	13	0	10
Ile	0	1	0	3	3
Leu	0	5	0	6	8
Lys	100	13	87	10	5
Met	0	1	0	0	6
Phe	0	1	0	4	3
Pro	0	3	0	3	7
Ser	0	15	0	11	9
Thr	0	1	0	15	5
Trp	0	1	0	2	1
Tyr	0	2	0	1	0
Val	0	7	0	3	1

Supplementary Table 4: Conservation of αCOPI in coronavirus zoonotic reservoirs and humans[1]

Organism	Yeast	Bat	Chicken	Pangolin	Camel	Human
**Yeast**	--	47.0/63.4	47.6/63.4	47.1/63.7	47.0/63.5	46.8/63.5
**Bat**	--	--	96.7/98.8	98.5/99.3	98.9/99.4	98.7/99.3
**Chicken**	--	--	--	96.5/98.7	96.7/98.9	96.7/98.6
**Pangolin**	--	--	--	--	99.1/99.8	98.5/99.6
**Camel**	--	--	--	--	--	98.9/99.8

[1]Conservation is represented as %identity/%similarity.
