# Supplementary material for: An extended motif in the SARS-CoV-2 spike modulates binding and release of host coatomer in retrograde trafficking
Source: Commun Biol. 2022 Feb 8;5:115. doi: 10.1038/s42003-022-03063-y (PMC8825798; doi:10.1038/s42003-022-03063-y)
Supplement: Supplementary file 2 — Description of Additional Supplementary Files [file 42003_2022_3063_MOESM2_ESM.pdf]

Description of Additional Supplementary Files

File name: Supplementary Data 1
Description: Multiple sequence alignment of αCOPI orthologs from the CONSURF server.

File name: Supplementary Data 2
Description: The table details the residue variety in % for each position in the query sequence. Each column shows the % for that amino-acid, found in position ('pos') in the MSA. In case there are residues which are not a standard amino-acid in the MSA, they are represented under column 'OTHER'. Gray and yellow are used to highlight Arg57, Asp115, and Tyr139.
